# Risk of subsequent primary neoplasms in survivors of adolescent and young adult cancer (Teenage and Young Adult Cancer Survivor Study): a population-based, cohort study

**DOI:** 10.1016/S1470-2045(18)30903-3

**Published:** 2019-04

**Authors:** Chloe J Bright, Raoul C Reulen, David L Winter, Daniel P Stark, Martin G McCabe, Angela B Edgar, Clare Frobisher, Michael M Hawkins

**Affiliations:** a Centre for Childhood Cancer Survivor Studies, Institute of Applied Health Research, University of Birmingham, Birmingham, UK; b Leeds Institute of Medical Research at St James's, School of Medicine, St James's University Hospital, Leeds, UK; c Division of Cancer Sciences, University of Manchester, Manchester Academic Health Science Centre, Manchester, UK; d Royal Hospital for Sick Children, Edinburgh, UK

## Abstract

**Background:**

Few studies have investigated the risks of subsequent primary neoplasms after adolescent and young adult (AYA) cancer. We investigated the risks of specific subsequent primary neoplasms after each of 16 types of AYA cancer.

**Methods:**

The Teenage and Young Adult Cancer Survivor Study is a population-based cohort of 200 945 survivors of cancer diagnosed when aged 15–39 years in England and Wales from Jan 1, 1971, to Dec 31, 2006. The cohort was established using cancer registrations from the Office for National Statistics and the Welsh Cancer registry. Follow-up was from 5-year survival until the first occurrence of death, emigration, or study end date (Dec 31, 2012). In this analysis, we focus on the risk of specific subsequent primary neoplasms after 16 types of AYA cancer: breast; cervical; testicular; Hodgkin lymphoma (female); Hodgkin lymphoma (male); melanoma; CNS (intracranial); colorectal; non-Hodgkin lymphoma; thyroid; soft-tissue sarcoma; ovarian; bladder; other female genital; leukaemia; and head and neck cancer. We report absolute excess risks (AERs; per 10 000 person-years) and cumulative incidence of specific types of subsequent primary neoplasm after each type of AYA cancer.

**Findings:**

During the 2 631 326 person-years of follow-up (median follow-up 16·8 years, IQR 10·5–25·2), 12 321 subsequent primary neoplasms were diagnosed in 11 565 survivors, most frequently among survivors of breast cancer, cervical cancer, testicular cancer, and Hodgkin lymphoma. AERs of any subsequent primary neoplasms were 19·5 per 10 000 person-years (95% CI 17·4–21·5) in survivors of breast cancer, 10·2 (8·0–12·4) in survivors of cervical cancer, 18·9 (16·6–21·1) in survivors of testicular cancer, 55·7 (50·4–61·1) in female survivors of Hodgkin lymphoma, and 29·9 (26·3–33·6) in male survivors of Hodgkin lymphoma. The cumulative incidence of all subsequent primary neoplasms 35 years after diagnosis was 11·9% (95% CI 11·3–12·6) in survivors of breast cancer, 15·8% (14·8–16·7) in survivors of cervical cancer, 20·2% (18·9–21·5) in survivors of testicular cancer, 26·6% (24·7–28·6) in female survivors of Hodgkin lymphoma, and 16·5% (15·2–18·0) in male survivors of Hodgkin lymphoma. In patients who had survived at least 30 years from diagnosis of cervical cancer, testicular cancer, Hodgkin lymphoma in women, breast cancer, and Hodgkin lymphoma in men, we identified a small number of specific subsequent primary neoplasms that account for 82%, 61%, 58%, 45%, and 41% of the total excess number of neoplasms, respectively. Lung cancer accounted for a notable proportion of the excess number of neoplasms across all AYA groups investigated.

**Interpretation:**

Our finding that a small number of specific subsequent primary neoplasms account for a large percentage of the total excess number of neoplasms in long-term survivors of cervical, breast, and testicular cancer, and Hodgkin lymphoma provides an evidence base to inform priorities for clinical long-term follow-up. The prominence of lung cancer after each of these AYA cancers indicates the need for further work aimed at preventing and reducing the burden of this cancer in future survivors of AYA cancer.

**Funding:**

Cancer Research UK, National Institute for Health Research, Academy of Medical Sciences, and Children with Cancer UK.

## Introduction

5-year relative survival after adolescent and young adult (AYA) cancer is 82% in Europe.^1^ Survivors are at increased risk of developing subsequent primary neoplasms, estimated to be between 1·5 and 3·1 times higher than that expected from the general population.^2^, ^3^

Previous large-scale studies of survivors of AYA cancers mostly concentrated on the risk of subsequent primary neoplasms after common cancers such as lymphomas, testicular cancer, and breast cancer.^4^, ^5^, ^6^, ^7^, ^8^, ^9^, ^10^, ^11^, ^12^, ^13^ Only one study comprehensively investigated the risks of developing any subsequent primary neoplasm after each type of AYA cancer.^3^ The main finding from the study was that survivors of AYA cancer had a higher absolute risk of developing a subsequent primary neoplasm than did survivors of childhood or adult cancer. Survivors of primary breast cancer and primary Hodgkin lymphoma had the highest absolute excess risk (54·4 and 48·6 per 10 000 person-years, respectively). However, the study did not investigate the risks of specific subsequent primary neoplasms after each specific type of AYA cancer.

**Research in context**

**Evidence before this study**

The risks of subsequent primary neoplasms in survivors of adolescent and young adult (AYA) cancers are largely unknown. We searched PubMed without any language or date restrictions using the keywords “teenage and young adult cancer OR adolescent and young adult cancer” and “survivor OR long-term” and “second cancer OR subsequent cancer” on Feb 16, 2016, for articles describing subsequent primary neoplasms in this population. A more focused search using each specific AYA cancer as keywords (eg, “Hodgkin lymphoma”) in place of “teenage and young adult cancer OR adolescent and young adult cancer” was also done. Additionally, we examined the bibliographies of selected references. Subsequent searches were done on Dec 31, 2016, and July 15, 2018. Most previous publications included the more common AYA cancers, but were not restricted to AYA cancer survivors. We identified only one study that investigated the risk of subsequent primary neoplasms after the entire spectrum of AYA cancers diagnosed at 15–39 years of age.

**Added value of this study**

To our knowledge, this is the largest study to investigate the risks of subsequent primary neoplasm after each specific AYA cancer and the first to provide excess risks of specific types of subsequent primary neoplasm after each of 16 types of AYA cancer. Unlike previous studies, which focused on the multiplicative risk, we concentrated on the absolute excess risk—ie, the excess number of subsequent primary neoplasms beyond those expected from the general population, which is directly interpretable in terms of adverse health impact on survivors. Our study shows that the excess number of subsequent primary neoplasms increased with increasing years from diagnosis for all AYA cancers investigated in detail. Additionally, we identified a small number of specific subsequent primary neoplasms that account for a substantial proportion of the total excess number of neoplasms in survivors of breast cancer, cervical cancer, testicular cancer, and Hodgkin lymphoma.

**Implications of all the available evidence**

Our findings advance previous knowledge on the risks of subsequent neoplasms after AYA cancer and provide an evidence base for identifying priorities for clinical follow-up of survivors of AYA cancer. Because lung cancer accounted for a notable proportion of the excess number of neoplasms across all AYA groups investigated, there is need for work aimed at reducing this burden among future survivors.

Evidence from previous studies of childhood cancer survivors suggests that the principal factors determining risks of subsequent primary neoplasms relate to aspects of treatments received for the original cancer.^14^, ^15^, ^16^ Treatment of AYA cancer varies greatly by cancer type and therefore, in the absence of detailed treatment information, it is essential to stratify risks by specific types of AYA cancer; such risk stratification provides an evidence base for clinical follow-up.

The aims of this large-scale population-based study were to calculate risks of all and specific subsequent primary neoplasms after each type of AYA cancer and to explore variation in these risks in relation to years from diagnosis, age at diagnosis, decade of diagnosis, and sex.

## Methods

### Study design and participants

The Teenage and Young Adult Cancer Survivor Study (TYACSS) was established using cancer registrations relating to neoplasms diagnosed between Jan 1, 1971, and Dec 31, 2006, in individuals aged 15–39 years inclusive, which were obtained from the Office for National Statistics for English cancer registrations, and the Welsh Cancer Intelligence and Surveillance Unit, Public Health Wales, for Welsh cancer registrations. Both tumour-related (eg, tumour site, morphology, date of diagnosis) and patient-related (eg, sex, date of birth, National Health Service [NHS] number, and unique patient identifier) information were obtained. The cancer registrations were checked for any errors, such as missing data in essential variables (including sex, date of birth, date of diagnosis, tumour site, and tumour histology) and incorrect chronology of events (birth, cancer, death). Cancer registrations were excluded if the neoplasm was not malignant (apart from intracranial, intraspinal, and bladder neoplasms where any behaviour was allowed), the histological type was not in the International Classification of Diseases for Oncology classification, the histological type was a non-melanoma skin cancer (these are underascertained by cancer registries), or if they were duplicate registrations. Individuals who had not survived 5 years from their first neoplasm were excluded. We also excluded individuals who had a previous childhood cancer included in the British Childhood Cancer Survivor Study.^17^ If an individual had multiple neoplasms diagnosed as an AYA cancer, then the first was regarded as the index cancer for the cohort. The process to create the cohort is described in the appendix (p 2). The resulting cohort of 200 945 5-year survivors of AYA cancer was linked to the national cancer and death registers via NHS Digital. This enabled ascertainment of all cancers and deaths for patients in the TYACSS cohort up to and including Dec 31, 2012. Ethical approval was provided by the National Research Ethics Service and permission to process information without individual consent by the National Information Governance Board for Health and Social Care.

### Procedures

Information on cancer diagnosis, sex, age at cancer diagnosis, decade of cancer diagnosis, and years since diagnosis were derived from the cancer registration information. Treatment information was not available because our sole source of data was national cancer registration. First primary neoplasms were grouped according to the internationally acknowledged classification scheme for tumours diagnosed in adolescence and young adulthood.^18^ Carcinomas and germ cell tumours were further subdivided by anatomical site because of the implications of radiotherapy site for the risk of subsequent primary neoplasm (appendix pp 3–5). We aimed to produce risk estimates after each specific first primary neoplasm; therefore, survivors of cancers categorised as “other” cancers were not included (appendix pp 2, 6). We focused on the risk of specific subsequent primary neoplasms after 16 types of AYA cancer: breast; cervical; testicular; Hodgkin lymphoma (female); Hodgkin lymphoma (male); melanoma; CNS (intracranial); colorectal; non-Hodgkin lymphoma; thyroid; soft-tissue sarcoma; ovarian; bladder; other female genital; leukaemia; and head and neck cancer. Age at diagnosis was categorised using 5-year age bands, which divided the total period equally (15–39 years). Decade of diagnosis was divided into 1971–79, 1980–89, and 1990–2006, in order to broadly describe differences in treatment given in these periods (early chemotherapy, chemotherapy, and modern treatment era). Years since diagnosis were classified using 10-year bands following diagnosis.

Individual patient record linkage to national cancer registries enabled data to be obtained indicating when an individual in the cohort had a subsequent cancer registration. Subsequent cancer registrations were classified as a subsequent primary neoplasm according to the International Association of Cancer Registries (IACR) and International Agency for Research on Cancer (IARC) rules for determining multiple primary tumours using the IACR/IARC Tools software. To reduce the likelihood of a local spread of the original AYA cancer being classified as a subsequent primary neoplasm, potential subsequent primary neoplasms occurring in anatomical sites close to the first primary neoplasm were excluded (appendix pp 7–8). Additionally, we excluded any cancer in a contralateral paired organ. Consequently, reported risk estimates are inevitably conservative.

### Statistical analysis

Individuals were followed from 5-year survival until the first occurrence of death, emigration, or study end date (Dec 31, 2012). Standardised incidence ratios (SIRs) were calculated as observed over expected numbers of neoplasms. Absolute excess risks (AERs) were calculated as the observed minus expected number of neoplasms, divided by the person-years at risk and multiplied by 10 000. The expected number of neoplasms was derived by multiplying the number of person-years accrued, stratified by sex, attained age (5-year bands), and calendar year (1-year bands) by the corresponding cancer rate in the general population of England and Wales,^19^ and summing appropriately. For AYA cancers with 200 or more observed subsequent primary neoplasms, SIRs are reported by specific type of subsequent primary neoplasm. We restrict attention to first primary neoplasm/subsequent primary neoplasm combinations with at least 100 subsequent primary neoplasms and a statistically significant SIR. We also identify those first primary neoplasm/subsequent primary neoplasm combinations with between 25 and 99 subsequent primary neoplasms and a statistically significant SIR of at least 5. We considered SIRs to be statistically significant at the 5% level (two-tailed test) if the 95% CI did not include 1. We considered AERs to be statistically significant at the 5% level (two-tailed test) if the 95% CI did not include 0.

AERs were stratified by years from diagnosis, age at diagnosis, decade of diagnosis, and sex where there were at least 100 subsequent primary neoplasms. To explore the simultaneous effect of these explanatory factors, multivariable Poisson regression incorporating the expected number of events was used to derive relative excess risks (RERs).^20^ RERs can be interpreted as the ratio of AERs adjusted for other potential explanatory factors included within the statistical model. The key assumption of Poisson regression (mean=variance) was met, and there was no evidence of overdispersion. AERs by an explanatory factor are reported if both the univariable and multivariable tests for linear trend in the AERs were each significant and the difference in the AERs between the lowest and highest level of the risk factor was at least nine excess subsequent primary neoplasms. A likelihood ratio test was used to test for linear trend in a factor by comparing the log-likelihood of a model including the variable of interest with the log-likelihood of a model without the variable of interest. Resulting p values are presented. Two-sided p values <0·05 were considered statistically significant. In deciding the percentage of the total AER attributable to specific subsequent primary neoplasms in relation to years from diagnosis, we ignored negative values for the AER and focused only on the positive values. Thus, the total AER (for the purposes of calculating percentages) after each specific first primary neoplasm is the sum of the positive values for the contributing subsequent primary neoplasms.

Cumulative incidence was calculated treating death as a competing risk. Multiple subsequent primary neoplasms within an individual were allowed for and counted in all analyses involving observed and expected numbers of subsequent primary neoplasms. This approach avoided bias because the expected numbers (based on cancer registrations in the general population) count all subsequent primary neoplasms. Analyses were done with Stata version 14.1.

We did sensitivity analyses in which subsequent leukaemia was included among survivors of AYA Hodgkin lymphoma, leukaemia, and non-Hodgkin lymphoma, and subsequent sarcoma was included among survivors of soft-tissue sarcoma and bone tumours.

### Role of the funding source

The funder of the study had no role in study design, data collection, data analysis, data interpretation, or writing of the report. CJB, RCR, DLW, and MMH had access to the raw data. The corresponding author had full access to all the data in the study and had final responsibility for the decision to submit for publication.

## Results

The TYACSS cohort comprises 200 945 5-year survivors of cancer diagnosed when aged 15–39 years, between Jan 1, 1971, and Dec 31, 2006, in England and Wales. Cohort characteristics are shown in table 1. 3118 individuals with “other” cancers were excluded from the analysis (appendix pp 2, 6). During the 2 631 326 person-years of follow-up (median follow-up 16·8 years, IQR 10·5–25·2), 12 321 subsequent primary neoplasms were diagnosed in 11 565 (6%) of the 197 827 survivors included in the analysis. Subsequent primary neoplasms were most frequently seen in survivors of breast cancer (1877 [15%] of 12 321 subsequent primary neoplasms), cervical cancer (1675 [14%]), Hodgkin lymphoma (1606 [13%]), and testicular cancer (1435 [12%]; table 1). Median follow-up was 14·3 years (IQR 9·1–22·3) in survivors of breast cancer, 20·2 years (12·8–27·2) in survivors of cervical cancer, 17·7 years (11·7–25·3) in survivors of testicular cancer, 19·3 years (12·3–27·0) in female survivors of Hodgkin lymphoma, and 19·6 years (12·1–27·5) in male survivors of Hodgkin lymphoma. Investigation of all first primary neoplasm/subsequent primary neoplasm combinations with at least 100 subsequent primary neoplasms showed that neither age at diagnosis nor decade of diagnosis was systematically associated with AERs, apart from age at diagnosis for breast cancer after female Hodgkin lymphoma and decade of diagnosis for lung cancer after male Hodgkin lymphoma (appendix p 9). Consequently, in this report we consider only variation of AERs with years from diagnosis and sex.

**Table 1:** Cohort characteristics

	**5-year survivors (n=200 945)**	**Subsequent primary neoplasms (n=12 321)**
**Sex**		
Male	76 666 (38%)	4282 (35%)
Female	124 279 (62%)	8039 (65%)
**Age at diagnosis of AYA cancer (years)**		
15–19	12 248 (6%)	622 (5%)
20–24	21 258 (11%)	991 (8%)
25–29	35 894 (18%)	1768 (14%)
30–34	54 541 (27%)	3333 (27%)
35–39	77 004 (38%)	5607 (46%)
**Decade of diagnosis of AYA cancer**		
1971–79	25 158 (13%)	4222 (34%)
1980–89	51 573 (26%)	5017 (41%)
1990–2006	124 214 (62%)	3082 (25%)
**AYA cancer**		
Breast	36 236 (18%)	1877 (15%)
Testicular	24 309 (12%)	1435 (12%)
Cervix	23 281 (12%)	1675 (14%)
Melanoma	22 446 (11%)	981 (8%)
Hodgkin lymphoma	16 971 (8%)	1606 (13%)
Non-Hodgkin lymphoma	9467 (5%)	511 (4%)
Thyroid	7809 (4%)	473 (4%)
CNS (intracranial)*	14 616 (7%)	739 (6%)
Colorectal	5805 (3%)	537 (4%)
Soft-tissue sarcoma	6130 (3%)	400 (3%)
Ovary	4885 (2%)	349 (3%)
Leukaemia	5073 (3%)	234 (2%)
Bladder	4685 (2%)	344 (3%)
Head and neck	3961 (2%)	253 (2%)
Lung	1219 (<1%)	80 (<1%)
Female genital (other)†	2270 (1%)	269 (2%)
Spinal cord and other CNS	2602 (1%)	191 (2%)
Other digestive	1419 (<1%)	107 (<1%)
Urinary (other)‡	1979 (1%)	139 (1%)
Bone tumour	2241 (1%)	100 (<1%)
Male genital (other)§	423 (<1%)	21 (<1%)
Other	3118 (2%)	..¶
**Time since diagnosis of AYA cancer (years)**		
5–9	46 679 (23%)	1863 (15%)
10–19	75 801 (38%)	4381 (36%)
20–29	50 793 (25%)	4285 (35%)
≥30	27 672 (14%)	1792 (15%)

Data are n (%). AYA=adolescent and young adult.

* CNS intracranial including brain, meninges, and pituitary gland.

† Female genital excluding cervix and ovary.

‡ Urinary excluding bladder.

§ Male genital excluding testis.

¶ Other AYA cancers were not included in the analysis of subsequent primary neoplasms.

Female survivors of breast cancer had an excess risk of developing any subsequent primary neoplasm corresponding to 20 excess subsequent primary neoplasms per 10 000 person-years (SIR 1·8, 95% CI 1·7–1·8; AER 19·5 per 10 000 person-years, 95% CI 17·4–21·5; table 2). SIRs for subsequent primary cancers of ovarian, lung, corpus uteri, other genital, melanoma, and colorectal sites were statistically significantly increased (table 3). The total AER of developing any subsequent primary neoplasm increased statistically significantly with time from breast cancer diagnosis to an AER of 25·6 per 10 000 person-years (95% CI 10·4–40·8) subsequent to 30 years from diagnosis (p<0·0001; table 4). Similarly, the AER for developing lung cancer after breast cancer increased statistically significantly with years from diagnosis (p<0·0001; table 4). In patients who had survived at least 30 years, the AER for lung cancer (13·1 per 10 000 person-years, 95% CI 5·1–21·0) accounted for 45% of the total number of excess neoplasms (total AER 28·9 per 10 000 person-years when negative AERs are excluded). The cumulative incidence of lung subsequent primary neoplasms at 35 years from diagnosis was 2·9% (95% CI 2·5–3·2), whereas an incidence of 2·0% was expected (figure, table 5).

**Table 2:** Risk of any subsequent primary neoplasm after specific types of adolescent and young adult cancer

	**Total number of subsequent primary neoplasms**	**Females**					**Males**				
		Survivors	Obs/exp	SIR (95% CI)	AER per 10 000 person-years (95% CI)	35-year cumulative incidence,% (95% CI)	Survivors	Obs/exp	SIR (95% CI)	AER per 10 000 person-years (95% CI)	35-year cumulative incidence,% (95% CI)
Breast	1877	36 236	1877/1069·6	1·8 (1·7–1·8)	19·5 (17·4 to 21·5)	11·9% (11·3–12·6)	..	..	..	..	..
Cervix	1675	23 281	1675/1307·0	1·3 (1·2–1·3)	10·2 (8·0 to 12·4)	15·8% (14·8–16·7)	..	..	..	..	..
Hodgkin lymphoma	1606	7422	903/288·2	3·1 (2·9–3·3)	55·7 (50·4 to 61·1)	26·6% (24·7–28·6)	9549	703/271·9	2·6 (2·4–2·8)	29·9 (26·3 to 33·6)	16·5% (15·2–18·0)
Testicular	1435	..	..	..	..	..	24 309	1435/807·6	1·8 (1·7–1·9)	18·9 (16·6 to 21·1)	20·2% (18·9–21·5)
Melanoma	981	15 212	751/665·7	1·1 (1·0–1·2)	4·5 (1·6 to 7·3)	13·2% (11·9–14·5)	7234	230/195·8	1·2 (1·0–1·3)	4·2 (0·6 to 7·9)	11·4% (9·4–16·6)
CNS (intracranial)*	739	7756	432/322·2	1·3 (1·2–1·5)	11·1 (7·0 to 15·2)	11·9% (10·6–13·3)	6860	307/184·5	1·7 (1·5–1·9)	14·8 (10·7 to 19·0)	10·0% (8·7–11·4)
Colorectal	537	2944	302/170·6	1·8 (1·6–2·0)	32·1 (23·8 to 40·4)	18·1% (15·8–20·5)	2861	235/125·0	1·9 (1·6–2·1)	28·9 (21·0 to 36·8)	19·5% (16·7–22·4)
Non-Hodgkin lymphoma	511	3719	216/149·5	1·4 (1·3–1·7)	14·8 (8·4 to 21·2)	15·5% (13·0–18·1)	5748	295/163·4	1·8 (1·6–2·0)	18·6 (13·8 to 23·4)	14·6% (12·6–16·7)
Thyroid	473	6215	397/288·8	1·4 (1·2–1·5)	13·1 (8·4 to 17·8)	18·2% (16·1–20·5)	1594	76/55·0	1·4 (1·1–1·7)	10·2 (1·9 to 18·4)	15·1% (10·6–20·4)
Soft-tissue sarcoma	400	3006	255/165·7	1·5 (1·4–1·7)	19·8 (12·9 to 26·8)	15·9% (13·7–18·2)	3124	145/106·5	1·4 (1·1–1·6)	9·3 (3·6 to 15·0)	11·8% (9·5–14·2)
Ovary	349	4885	349/255·3	1·4 (1·2–1·5)	12·3 (7·5 to 17·1)	13·9% (12·2–15·7)	..	..	..	..	..
Bladder	344	1257	88/87·9	1·0 (0·8–1·2)	0·0 (−8·9 to 8·9)	14·3% (10·9–18·0)	3428	256/209·2	1·2 (1·1–1·4)	8·0 (2·6 to 13·4)	13·9% (12·0–15·8)
Female genital (other)†	269	2270	269/141·8	1·9 (1·7–2·1)	37·2 (27·8 to 46·7)	20·9% (18·1–23·8)	..	..	..	..	..
Leukaemia	234	2187	120/63·3	1·9 (1·6–2·3)	22·9 (14·2 to 31·5)	15·9% (12·2–19·9)	2886	114/44·4	2·6 (2·1–3·1)	22·7 (15·9 to 29·5)	15·0% (11·2–19·4)
Head and neck	253	1744	117/87·9	1·3 (1·1–1·6)	12·4 (3·4 to 21·5)	13·5% (10·8–16·4)	2217	136/100·3	1·4 (1·1–1·6)	11·6 (4·2 to 19·0)	13·0% (10·6–15·6)
Spinal cord and other CNS	191	1255	95/62·5	1·5 (1·2–1·9)	19·1 (7·9 to 30·3)	17·3% (12·8–22·2)	1347	96/44·0	2·2 (1·8–2·7)	30·6 (19·3 to 41·9)	19·2% (14·7–24·2)
Urinary (other)‡	139	801	58/39·0	1·5 (1·1–1·9)	19·1 (4·1 to 34·1)	15·1% (10·7–20·2)	1178	81/45·7	1·8 (1·4–2·2)	24·5 (12·2 to 36·7)	18·6% (13·8–24·0)
Other digestive	107	638	45/30·3	1·5 (1·1–2·0)	19·3 (2·0 to 36·6)	12·5% (8·4–17·3)	781	62/29·5	2·1 (1·6–2·7)	33·9 (17·8 to 50·1)	14·5% (10·4–19·1)
Bone tumour	100	957	52/32·7	1·6 (1·2–2·1)	14·7 (3·9 to 25·5)	12·1% (8·5–16·5)	1284	48/33·1	1·4 (1·1–1·9)	8·1 (0·7 to 15·6)	8·8% (6·0–12·4)
Lung	80	563	38/30·0	1·3 (0·9–1·7)	10·2 (−5·2 to 25·6)	11·0% (7·1–15·9)	656	42/37·4	1·1 (0·8–1·5)	4·4 (−7·8 to 16·7)	11·8% (8·3–16·0)
Male genital (other)§	21	..	..	..	..	..	423	21/16·8	1·2 (0·8–1·9)	7·2 (−8·2 to 22·5)	13·5% (7·4–21·5)

AER=absolute excess risk. Obs/exp=observed number of subsequent primary neoplasms/expected number of subsequent primary neoplasms. SIR=standardised incidence ratio. ..=not applicable.

* CNS intracranial including brain, meninges, and pituitary gland.

† Female genital excluding cervix and ovary.

‡ Urinary excluding bladder.

§ Male genital excluding testis.

**Table 3:** Risk of specific subsequent primary neoplasms (row headings) after specific AYA cancers* (column headings) with at least 200 subsequent primary neoplasms observed in total

	**All**†		**Breast**		**Cervix**		**Testicular**		**Hodgkin lymphoma (female)**		**Hodgkin lymphoma (male)**		**Melanoma**		**CNS (intracranial)**		**Colorectal**		**Non-Hodgkin lymphoma**		**Thyroid**		**Soft-tissue sarcoma**		**Ovary**		**Bladder**		**Female genital(other)**		**Leukaemia**		**Head and neck**	
	Obs/exp	SIR (95% CI)	Obs/exp	SIR (95% CI)	Obs/exp	SIR (95% CI)	Obs/exp	SIR (95% CI)	Obs/exp	SIR (95% CI)	Obs/exp	SIR (95% CI)	Obs/exp	SIR (95% CI)	Obs/exp	SIR (95% CI)	Obs/exp	SIR (95% CI)	Obs/exp	SIR (95% CI)	Obs/exp	SIR (95% CI)	Obs/exp	SIR (95% CI)	Obs/exp	SIR (95% CI)	Obs/exp	SIR (95% CI)	Obs/exp	SIR (95% CI)	Obs/exp	SIR (95% CI)	Obs/exp	SIR (95% CI)
Breast	2260/2032·1	1·1 (1·1–1·2)	..	..	532/684·8‡	0·8 (0·7–0·8)‡	..	..	431/136·3‡	3·2 (2·9–3·5)‡	..	..	359/313·2	1·1 (1·0–1·3)	100/145·6‡	0·7 (0·6–0·8)‡	74/79·3	0·9 (0·7–1·2)	89/70·9	1·3 (1·0–1·5)	170/130·2‡	1·3 (1·1–1·5)‡§	88/72·5	1·2 (1·0–1·5)	101/131·1‡	0·8 (0·6–0·9)‡	29/39·3	0·7 (0·5–1·1)	80/71·2	1·1 (0·9–1·4)	50/30·3	1·6 (1·2–2·2)	44/39·2	1·1 (0·8–1·5)
Lung and bronchus	1740/855·8	2·0 (1·9–2·1)	357/152·2‡	2·3 (2·1–2·6)‡	335/116·9‡	2·9 (2·6–3·2)‡	171/115·5‡	1·5 (1·3–1·7)‡	101/19·0‡	5·3 (4·3–6·5)‡	198/41·3‡	4·8 (4·1–5·5)‡	82/78·1	1·1 (0·8–1·3)	34/47·5	0·7 (0·5–1·0)	48/38·1	1·3 (0·9–1·7)	83/37·0	2·2 (1·8–2·8)	34/28·4	1·2 (0·8–1·7)	32/27·7	1·2 (0·8–1·6)	54/23·2	2·3 (1·7–3·0)	73/45·2	1·6 (1·3–2·0)	29/14·2	2·0 (1·4–2·9)	14/9·9	1·4 (0·8–2·4)	41/23·0	1·8 (1·3–2·4)
Colorectal	1290/801·9	1·6 (1·5–1·7)	179/150·2‡	1·2 (1·0–1·4)‡¶	237/115·1‡	2·1 (1·8–2·3)‡	206/109·2‡	1·9 (1·6–2·2)‡	50/20·2	2·5 (1·8–3·3)	86/39·2	2·2 (1·8–2·7)	80/77·5	1·0 (0·8–1·3)	50/46·8	1·1 (0·8–1·4)	..	..	47/35·4	1·3 (1·0–1·8)	33/28·2	1·2 (0·8–1·6)	46/26·6	1·7 (1·3–2·3)	58/23·0	2·5 (1·9–3·3)	39/39·8	1·0 (0·7–1·3)	65/13·7	4·7 (3·6–6·0)	16/10·5	1·5 (0·9–2·5)	23/21·2	1·1 (0·7–1·6)
Other	793/405·1	2·0 (1·8–2·1)	97/56·7	1·7 (1·4–2·1)	61/46·6	1·3 (1·0–1·6)	104/52·4‡	2·0 (1·6–2·4)‡	37/6·4	5·8 (4·1–8·0)	54/27·7	1·9 (1·5–2·5)	64/42·4	1·5 (1·1–1·9)	96/31·5	3·1 (2·5–3·7)	31/17·1	1·8 (1·2–2·5)	33/16·7	2·0 (1·3–2·7)	33/15·2	2·2 (1·4–2·9)	19/14·1	1·3 (0·7–2)	19/9·4	2·0 (1·1–2·9)	26/19·1	1·4 (0·8–1·9)	15/5·2	2·9 (1·4–4·3)	15/7·3	2·1 (1·0–3·1)	28/11·6	2·4 (1·5–3·3)
Bladder	606/296·6	2·0 (1·9–2·2)	50/38·6	1·3 (1·0–1·7)	126/29·6‡	4·3 (3·5–5·1)‡	167/61·3‡	2·7 (2·3–3·2)‡	11/4·9	2·2 (1·1–4·0)	30/22·0	1·4 (0·9–1·9)	27/27·8	1·0 (0·6–1·4)	22/19·2	1·1 (0·7–1·7)	32/15·5	2·1 (1·4–2·9)	33/16·5	2·0 (1·4–2·8)	11/9·4	1·2 (0·6–2·1)	13/11·2	1·2 (0·6–2·0)	24/6·0	4·0 (2·6–6·0)	..	..	20/3·6	5·5 (3·4–8·5)	8/4·4	1·8 (0·8–3·6)	14/10·1	1·4 (0·8–2·3)
Prostate	545/433·5	1·3 (1·2–1·4)	..	..	..	..	185/133·5‡	1·4 (1·2–1·6)‡	..	..	48/45·8	1·0 (0·8–1·4)	40/32·9	1·2 (0·9–1·7)	34/29·0	1·2 (0·8–1·6)	33/27·3	1·2 (0·8–1·7)	35/29·8	1·2 (0·8–1·6)	14/9·1	1·5 (0·8–2·6)	24/17·8	1·4 (0·9–2·0)	..	..	62/46·4	1·3 (1·0–1·7)	..	..	5/6·1	0·8 (0·3–1·9)	15/19·6	0·8 (0·4–1·3)
Melanoma	500/385·6	1·3 (1·2–1·4)	100/81·0‡	1·2 (1·0–1·5)‡‖	61/68·3	0·9 (0·7–1·1)	68/44·4	1·5 (1·2–1·9)	29/17·1	1·7 (1·1–2·4)	20/16·8	1·2 (0·7–1·8)	..	..	37/26·8	1·4 (1·0–1·9)	20/13·7	1·5 (0·9–2·3)	27/17·2	1·6 (1·0–2·3)	25/17·4	1·4 (0·9–2·1)	24/13·2	1·8 (1·2–2·7)	19/13·6	1·4 (0·8–2·2)	14/13·1	1·1 (0·6–1·8)	6/6·6	0·9 (0·3–2·0)	12/7·3	1·6 (0·8–2·9)	††	††
Ovary	447/244·6	1·8 (1·7–2·0)	291/104·9‡	2·8 (2·5–3·1)‡	..	..	..	..	16/16·4	1·0 (0·6–1·6)	..	..	32/37·7	0·8 (0·6–1·2)	20/17·5	1·1 (0·7–1·8)	19/10·2	1·9 (1·1–2·9)	11/8·6	1·3 (0·6–2·3)	16/15·8	1·0 (0·6–1·6)	18/9·1	2·0 (1·2–3·1)	..	..	††	††	..	..	††	††	††	††
Oral	406/215·3	1·9 (1·7–2·1)	44/28·3	1·6 (1·1–2·1)	42/23·2	1·8 (1·3–2·4)	56/40·7	1·4 (1·0–1·8)	21/4·6	4·6 (2·8–7·0)	51/14·8	3·5 (2·6–4·5)	13/20·8	0·6 (0·3–1·1)	18/13·9	1·3 (0·8–2·0)	16/8·8	1·8 (1·0–2·9)	31/11·0	2·8 (1·9–4·0)	13/7·0	1·9 (1·0–3·2)	16/7·5	2·1 (1·2–3·5)	10/4·5	2·2 (1·1–4·1)	12/10·9	1·1 (0·6–1·9)	7/2·4	2·9 (1·2–5·9)	29/3·7‡‡	7·8 (5·2–11·1)‡‡	..	..
Corpus uteri	404/214·8	1·9 (1·7–2·1)	204/95·7‡	2·1 (1·8–2·4)‡	..	..	..	..	21/12·8	1·6 (1·0–2·5)	..	..	33/32·6	1·0 (0·7–1·4)	26/14·6	1·8 (1·2–2·6)	68/9·5‡‡	7·2 (5·6–9·1)‡‡	8/7·3	1·1 (0·5–2·2)	11/13·5	0·8 (0·4–1·5)	11/8·0	1·4 (0·7–2·5)	..	..	††	††	..	..	††	††	7/4·3	1·6 (0·7–3·4)
Kidney	371/175·8	2·1 (1·9–2·3)	39/27·9	1·4 (1·0–1·9)	32/22·2	1·4 (1·0–2·0)	53/30·4	1·7 (1·3–2·3)	11/4·0	2·7 (1·4–4·9)	23/11·0	2·1 (1·3–3·1)	31/17·4	1·8 (1·2–2·5)	73/11·2‡‡	6·5 (5·1–8·2)‡‡	23/7·7	3·0 (1·9–4·5)	11/8·8	1·3 (0·6–2·2)	10/6·0	1·7 (0·8–3·0)	14/6·2	2·3 (1·2–3·8)	6/4·3	1·4 (0·5–3·0)	..	..	††	††	7/2·8	2·5 (1·0–5·2)	13/4·9	2·6 (1·4–4·5)
Non-Hodgkin lymphoma	358/290·4	1·2 (1·1–1·4)	79/57·2	1·4 (1·1–1·7)	51/46·0	1·1 (0·8–1·5)	63/46·2	1·4 (1·0–1·7)	..	..	..	..	41/32·1	1·3 (0·9–1·7)	25/20·1	1·2 (0·8–1·8)	19/12·7	1·5 (0·9–2·3)	..	..	13/11·6	1·1 (0·6–1·9)	13/10·8	1·2 (0·6–2·1)	8/9·1	0·9 (0·4–1·7)	11/14·0	0·8 (0·4–1·4)	6/5·1	1·2 (0·4–2·6)	..	..	9/8·0	1·1 (0·5–2·1)
Brain	317/186·7	1·7 (1·5–1·9)	28/29·4	1·0 (0·6–1·4)	22/24·0	0·9 (0·6–1·4)	26/28·9	0·9 (0·6–1·3)	11/5·4	2·0 (1·0–3·6)	16/11·4	1·4 (0·8–2·3)	45/18·4	2·4 (1·8–3·3)	34/6·8‡‡	5·0 (3·5–7·0)‡‡	21/7·0	3·0 (1·8–4·6)	9/8·8	1·0 (0·5–1·9)	13/6·6	2·0 (1·0–3·4)	13/6·3	2·1 (1·1–3·5)	††	††	8/8·0	1·0 (0·4–2·0)	5/2·6	1·9 (0·6–4·5)	17/3·3	5·1 (3·0–8·2)	9/4·6	2·0 (0·9–3·7)
Oesophagus	297/158·4	1·9 (1·7–2·1)	46/20·4	2·3 (1·6–3·0)	23/15·6	1·5 (0·9–2·2)	46/29·8	1·5 (1·1–2·1)	26/2·6‡‡	10·2 (6·6–14·9)‡‡	46/10·7	4·3 (3·2–5·8)	14/14·1	1·0 (0·5–1·7)	6/9·5	0·6 (0·2–1·4)	12/7·3	1·6 (0·8–2·9)	20/8·0	2·5 (1·5–3·9)	5/4·7	1·1 (0·3–2·5)	5/5·5	0·9 (0·3–2·1)	††	††	7/9·6	0·7 (0·3–1·5)	5/1·9	2·6 (0·9–6·1)	14/2·2	6·3 (3·4–10·6)	13/4·8	2·7 (1·5–4·7)
Pancreas	283/162·8	1·7 (1·5–2·0)	34/30·3	1·1 (0·8–1·6)	26/23·0	1·1 (0·7–1·7)	76/20·9	3·6 (2·9–4·6)	13/3·8	3·4 (1·8–5·8)	22/7·5	2·9 (1·8–4·4)	19/15·1	1·3 (0·8–2·0)	22/9·0	2·4 (1·5–3·7)	9/6·9	1·3 (0·6–2·5)	8/6·8	1·2 (0·5–2·3)	6/5·5	1·1 (0·4–2·4)	††	††	9/4·6	1·9 (0·9–3·7)	11/7·8	1·4 (0·7–2·5)	5/2·8	1·8 (0·6–4·1)	††	††	††	††
Other female genital	282/219·7	1·3 (1·1–1·4)	111/82·0‡	1·4 (1·1–1·6)‡	..	..	..	..	31/19·8	1·6 (1·1–2·2)	..	..	25/35·9	0·7 (0·5–1·0)	22/18·1	1·2 (0·8–1·8)	27/8·1	3·3 (2·2–4·8)	13/8·4	1·5 (0·8–2·6)	16/15·5	1·0 (0·6–1·7)	8/8·6	0·9 (0·4–1·8)	..	..	7/4·1	1·7 (0·7–3·5)	..	..	9/4·2	2·1 (1·0–4·0)	7/4·4	1·6 (0·6–3·3)
Stomach	264/147·6	1·8 (1·6–2·0)	32/20·4	1·6 (1·1–2·2)	22/15·2	1·4 (0·9–2·2)	81/25·7	3·2 (2·5–3·9)	7/2·8	2·5 (1·0–5·2)	26/9·4	2·8 (1·8–4·1)	7/13·1	0·5 (0·2–1·1)	6/8·8	0·7 (0·3–1·5)	14/6·9	2·0 (1·1–3·4)	18/7·3	2·5 (1·5–3·9)	10/4·5	2·2 (1·1–4·1)	13/5·1	2·6 (1·4–4·4)	††	††	12/9·0	1·3 (0·7–2·3)	††	††	††	††	††	††
Other digestive§§	254/89·7	2·8 (2·5–3·2)	47/18·3	2·6 (1·9–3·4)	31/14·3	2·2 (1·5–3·1)	39/9·6	4·1 (2·9–5·6)	9/2·5	3·5 (1·6–6·7)	12/3·5	3·4 (1·8–6·0)	12/8·7	1·4 (0·7–2·4)	8/5·0	1·6 (0·7–3·2)	48/3·4‡‡	13·9 (10·3–18·4)‡‡	††	††	9/3·2	2·8 (1·3–5·3)	11/2·8	4·0 (2·0–7·1)	8/2·8	2·8 (1·2–5·6)	††	††	6/1·7	3·6 (1·3–7·9)	††	††	††	††
Thyroid	244/90·8	2·7 (2·4–3·0)	35/20·4	1·7 (1·2–2·4)	20/17·5	1·1 (0·7–1·8)	13/5·4	2·4 (1·3–4·1)	47/5·1‡‡	9·2 (6·7–12·2)‡‡	26/2·1‡‡	12·3 (8·0–18·0)‡‡	21/11·0	1·9 (1·2–2·9)	11/6·0	1·8 (0·9–3·3)	††	††	12/3·4	3·6 (1·8–6·3)	..	..	9/2·8	3·3 (1·5–6·2)	9/3·6	2·5 (1·1–4·8)	††	††	5/1·6	3·1 (1·0–7·2)	10/1·7	6·0 (2·9–11·0)	7/1·7	4·2 (1·7–8·7)
Leukaemia	229/151·9	1·5 (1·3–1·7)	51/28·4	1·8 (1·3–2·4)	28/22·7	1·2 (0·8–1·8)	39/25·5	1·5 (1·1–2·1)	..	..	..	..	15/16·7	0·9 (0·5–1·5)	23/10·8	2·1 (1·4–3·2)	9/6·9	1·3 (0·6–2·5)	..	..	10/6·1	1·7 (0·8–3·0)	9/5·8	1·6 (0·7–3·0)	10/4·6	2·2 (1·1–4·0)	11/8·0	1·4 (0·7–2·5)	††	††	..	..	††	††
Other respiratory	217/92·8	2·3 (2·0–2·7)	30/10·4	2·9 (1·9–4·1)	18/8·2	2·2 (1·3–3·5)	31/19·4	1·6 (1·1–2·3)	20/1·5	13·1 (8·0–20·3)	42/7·2‡‡	5·9 (4·2–7·9)‡‡	12/8·4	1·4 (0·7–2·5)	12/6·0	2·0 (1·0–3·5)	8/4·4	1·8 (0·8–3·6)	12/5·1	2·4 (1·2–4·1)	9/2·8	3·2 (1·5–6·1)	††	††	††	††	9/6·0	1·5 (0·7–2·9)	††	††	††	††	††	††
Meninges	214/70·8	3·0 (2·6–3·5)	23/16·9	1·4 (0·9–2·0)	8/13·8	0·6 (0·3–1·1)	11/4·2	2·6 (1·3–4·7)	11/3·0	3·7 (1·8–6·5)	††	††	9/7·6	1·2 (0·5–2·2)	60/3·1‡‡	19·1 (14·6–24·6)‡‡	††	††	7/2·4	2·9 (1·2–6·0)	12/3·0	4·0 (2·0–6·9)	6/2·0	3·0 (1·1–6·5)	6/2·7	2·2 (0·8–4·9)	††	††	††	††	13/1·0	13·2 (7·0–22·6)	††	††

AYA=adolescent and young adult. Obs/exp=observed number of subsequent primary neoplasms/expected number of subsequent primary neoplasms. SIR=standardised incidence ratio.

* Excludes potential subsequent primary neoplasms at the same anatomical site as the AYA cancer, represented by …

† Includes all first primary neoplasms listed and other male genital and bone tumours.

‡ At least 100 observed events and significant SIR.

§ Breast subsequent primary neoplasms after thyroid cancer occurred only in females; expected breast cancers in males was zero.

¶ Confidence interval to three decimal places was 1·023–1·380.

‖ Confidence interval to three decimal places was 1·004–1·501.

†† Results not reliable because of small number of subsequent primary neoplasms (<5 observed subsequent primary neoplasms).

‡‡ Between 25 and 99 observed events, SIR at least 5.

§§ Consists of 80 small intestine, 62 gallbladder, 77 retroperitoneum and peritoneum, and 31 other or unspecified.

**Table 4:** AER of all and specific subsequent primary neoplasms after specific first primary neoplasm by time from diagnosis, with percentage of total AER contributed by specific subsequent primary neoplasms

		**5–9 years**			**10–19 years**			**20–29 years**			**≥30 years**			**p value**†
		Obs/exp	AER per 10 000 person-years (95% CI)	% of total AER*	Obs/exp	AER per 10 000 person-years (95% CI)	% of total AER*	Obs/exp	AER per 10 000 person-years (95% CI)	% of total AER*	Obs/exp	AER per 10 000 person-years (95% CI)	% of total AER*	
**First primary neoplasm: female breast**														
Total subsequent primary neoplasms‡		371/190·4	11·7 (9·3 to 14·2)	100%	730/391·7	19·6 (16·5 to 22·6)	100%	581/338·5	34·5 (27·8 to 41·2)	100%	195/149·0	25·6 (10·4 to 40·8)	100%	<0·0001
	Corpus uteri	46/9·9	2·3 (1·5 to 3·2)	19·7%	100/36·0	3·7 (2·6 to 4·8)	18·9%	51/36·8	2·0 (0·0 to 4·0)	5·8%	7/12·9	−3·3 (−6·2 to −0·4)	..§	0·97
	Ovary	74/20·6	3·5 (2·4 to 4·6)	29·9%	128/43·0	4·9 (3·6 to 6·2)	25·0%	71/31·2	5·7 (3·3 to 8·0)	16·4%	18/10·1	4·4 (−0·2 to 9·0)	15·2%	0·50
	Other female genital	38/30·9	0·5 (−0·3 to 1·2)	4·3%	50/33·1	1·0 (0·2 to 1·8)	5·1%	18/13·7	0·6 (−0·6 to 1·8)	1·7%	5/4·3	0·4 (−2·1 to 2·8)	1·4%	0·25
	Colorectal	24/19·8	0·3 (−0·3 to 0·9)	2·6%	63/51·6	0·7 (−0·2 to 1·6)	3·6%	65/51·9	1·9 (−0·4 to 4·1)	5·5%	27/26·9	0·1 (−5·6 to 5·7)	0·3%	0·13
	Lung	37/14·6	1·5 (0·7 to 2·2)	12·8%	112/48·5	3·7 (2·5 to 4·9)	18·9%	154/58·6	13·6 (10·1 to 17·0)	39·2%	54/30·5	13·1 (5·1 to 21·0)	45·2%	<0·0001
	Melanoma	30/23·1	0·4 (−0·3 to 1·1)	3·4%	45/34·0	0·6 (−0·1 to 1·4)	3·1%	17/18·1	−0·2 (−1·3 to 1·0)	..§	8/5·8	1·2 (−1·9 to 4·3)	4·1%	0·57
	Other	122/71·5	3·3 (1·9 to 4·7)	28·2%	232/145·5	5·0 (3·3 to 6·7)	25·5%	205/128·2	10·9 (6·9 to 14·9)	31·4%	76/58·4	9·8 (0·3 to 19·3)	33·8%	0·0001
**First primary neoplasm: cervix**														
Total subsequent primary neoplasms¶		241/179·6	5·7 (2·9 to 8·5)	100%	618/509·3	6·9 (3·8 to 10·0)	100%	609/465·2	18·3 (12·2 to 24·5)	100%	207/152·9	32·3 (15·4 to 49·1)	100%	<0·0001
	Breast	89/104·4	−1·4 (−3·1 to 0·3)	..§	251/294·6	−2·8 (−4·7 to −0·8)	..§	157/227·0	−8·9 (−12·1 to −5·8)	..§	35/58·8	−14·2 (−21·1 to −7·3)	..§	..‖
	Bladder	11/2·1	0·8 (0·2 to 1·4)	11·3%	40/8·4	2·0 (1·2 to 2·8)	20·6%	52/12·9	5·0 (3·2 to 6·8)	18·3%	23/6·1	10·1 (4·5 to 15·7)	21·7%	<0·0001
	Colorectal	23/10·4	1·2 (0·3 to 2·0)	16·9%	66/37·9	1·8 (0·8 to 2·8)	18·6%	110/46·7	8·1 (5·4 to 10·7)	29·7%	38/20·0	10·7 (3·5 to 17·9)	23·0%	<0·0001
	Lung	45/7·3	3·5 (2·3 to 4·7)	49·3%	101/34·5	4·2 (3·0 to 5·5)	43·3%	137/52·0	10·8 (7·9 to 13·8)	39·6%	52/23·2	17·2 (8·8 to 25·6)	37·0%	<0·0001
	Other	73/55·3	1·6 (0·1 to 3·2)	22·5%	160/133·9	1·7 (0·1 to 3·2)	17·5%	153/126·6	3·4 (0·3 to 6·5)	12·5%	59/44·8	8·5 (−0·5 to 17·4)	18·3%	0·24
**First primary neoplasm: testicular**														
Total subsequent primary neoplasms**		124/81·9	3·8 (1·8 to 5·7)	100%	378/246·2	9·0 (6·4 to 11·6)	100%	605/318·3	46·6 (38·8 to 54·4)	100%	328/161·1	127·0 (100·0 to 154·0)	100%	<0·0001
	Prostate	††	††	††	26/20·0	0·4 (−0·3 to 1·1)	4·4%	79/66·3	2·1 (−0·8 to 4·9)	4·5%	79/45·7	25·3 (12·1 to 38·6)	19·9%	<0·0001
	Bladder	9/5·0	0·4 (−0·2 to 0·9)	10·5%	30/17·4	0·9 (0·1 to 1·6)	10·0%	84/25·0	9·6 (6·7 to 12·5)	20·6%	44/14·0	22·8 (13·0 to 32·7)	18·0%	<0·0001
	Colorectal	16/9·4	0·6 (−0·1 to 1·3)	15·8%	45/33·4	0·8 (−0·1 to 1·7)	8·9%	97/44·1	8·6 (5·5 to 11·7)	18·5%	48/22·3	19·6 (9·2 to 29·9)	15·4%	<0·0001
	Lung	7/7·1	−0·0 (−0·5 to 0·5)	0·0%	42/32·0	0·7 (−0·2 to 1·6)	7·8%	83/49·9	5·4 (2·5 to 8·3)	11·6%	39/26·6	9·5 (0·1 to 18·8)	7·5%	<0·0001
	Other	91/59·1	2·8 (1·2 to 4·5)	73·7%	235/143·4	6·3 (4·2 to 8·3)	70·0%	262/133·0	21·0 (15·8 to 26·1)	45·1%	118/52·6	49·7 (33·5 to 65·9)	39·1%	<0·0001
**First primary neoplasm: female Hodgkin lymphoma**														
Total subsequent primary neoplasms‡‡		66/38·6	8·0 (3·4 to 12·7)	100%	316/104·2	44·7 (37·4 to 52·1)	100%	374/100·8	119·5 (102·9 to 136·1)	100%	147/44·6	168·6 (129·5 to 207·8)	100%	<0·0001
	Breast	20/17·0	0·9 (−1·7 to 3·5)	11·3%	168/52·0	24·5 (19·1 to 29·9)	54·8%	181/48·8	57·8 (46·3 to 69·3)	48·4%	62/18·4	71·8 (46·4 to 97·2)	42·6%	<0·0001
	Lung	7/1·0	1·8 (0·2 to 3·3)	22·5%	25/4·8	4·3 (2·2 to 6·3)	9·6%	48/8·0	17·5 (11·6 to 23·5)	14·6%	21/5·2	26·0 (11·2 to 40·8)	15·4%	<0·0001
	Other	39/20·6	5·4 (1·8 to 9·0)	67·5%	123/47·4	16·0 (11·4 to 20·6)	35·8%	145/44·0	44·2 (33·9 to 54·5)	37·0%	64/21·0	70·8 (44·9 to 96·6)	42·0%	<0·0001
**First primary neoplasm: male Hodgkin lymphoma**														
Total subsequent primary neoplasms§§		51/25·1	5·9 (2·7 to 9·1)	100%	192/72·9	19·5 (15·0 to 23·9)	100%	289/105·1	60·2 (49·3 to 71·1)	100%	171/68·8	121·9 (91·3 to 152·4)	100%	<0·0001
	Lung	6/2·1	0·9 (−0·2 to 2·0)	15·3%	56/9·8	7·5 (5·2 to 9·9)	38·8%	82/17·5	21·1 (15·3 to 26·9)	35·0%	54/11·9	50·2 (33·0 to 67·3)	41·2%	<0·0001
	Other	45/23·0	5·0 (2·0 to 8·0)	84·7%	136/63·1	11·9 (8·2 to 15·7)	61·3%	207/87·7	39·1 (29·8 to 48·3)	65·0%	117/56·9	71·7 (46·4 to 97·0)	58·8%	<0·0001
**First primary neoplasm: female thyroid**														
Total subsequent primary neoplasms¶¶		61/47·0	5·0 (−0·5 to 10·5)	100%	155/107·8	13·6 (6·6 to 20·6)	100%	133/95·1	23·9 (9·7 to 38·1)	100%	48/38·9	21·9 (−10·9 to 54·7)	100%	0·03
Breast		27/22·1	1·7 (−1·9 to 5·4)	34·0%	63/53·2	2·8 (−1·7 to 7·3)	20·8%	59/41·2	11·2 (1·7 to 20·7)	46·9%	21/13·7	17·6 (−4·1 to 39·3)	80·4%	0·06
Other		34/24·9	3·3 (−0·8 to 7·3)	66·0%	92/54·6	10·7 (5·3 to 16·1)	79·2%	74/53·9	12·7 (2·1 to 23·3)	53·1%	27/25·2	4·3 (−20·2 to 28·9)	19·6%	0·12

AER=absolute excess risk. Obs/exp=observed number of subsequent primary neoplasms/expected number of subsequent primary neoplasms.

* The total AER (for the purposes of calculating percentages) after each specific first primary neoplasm is the sum of the positive values for the contributing subsequent primary neoplasms.

† Multivariable p value.

‡ All subsequent primary neoplasms in female survivors excluding subsequent primary neoplasms of the breast.

§ Negative numbers for the AER, represented by …

¶ All subsequent primary neoplasms in female survivors excluding subsequent primary neoplasms of female genital sites.

‖ p value not calculated due to negative AERs for all years.

** All subsequent primary neoplasms in male survivors excluding subsequent primary neoplasms of other male genital sites (prostate sites allowed).

†† Results not reliable because of small number of subsequent primary neoplasms (<5 observed subsequent primary neoplasms).

‡‡ All solid subsequent primary neoplasms in female survivors (excluding non-solid tumours).

§§ All solid subsequent primary neoplasms in male survivors (excluding non-solid tumours).

¶¶ All subsequent primary neoplasms in female survivors excluding subsequent primary neoplasms of the thyroid.

**Figure:**
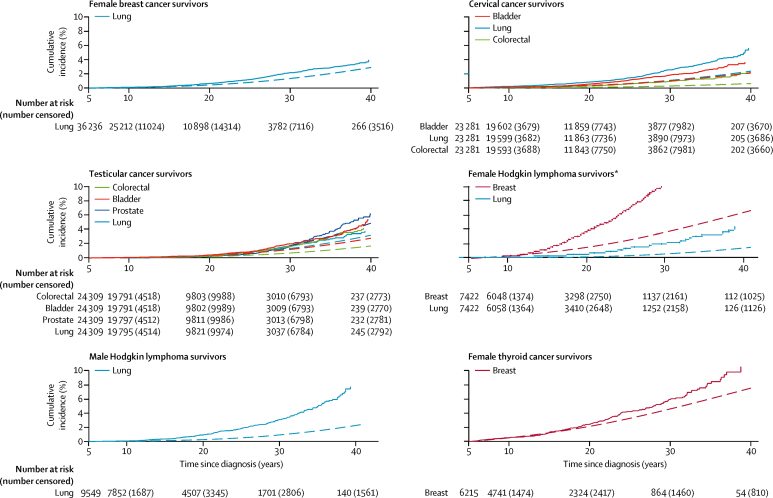
Observed (solid) and expected (dashed) cumulative incidence of specific subsequent primary neoplasms in survivors of adolescent and young adult cancer *Cumulative incidence exceeds scale; please refer to table 5 for cumulative incidence up to 35 years from diagnosis.

**Table 5:** Cumulative incidence of specific subsequent primary neoplasms after specific first primary neoplasms by years from diagnosis

		**10 years**	**15 years**	**20 years**	**25 years**	**30 years**	**35 years**
**First primary neoplasm: female breast**							
Total subsequent primary neoplasms*		1·1% (1·0–1·2)	2·5% (2·3–2·7)	4·2% (4·0–4·5)	6·4% (6·1–6·8)	9·3% (8·8–9·8)	11·9% (11·3–12·6)
	Corpus uteri	0·1% (0·1–0·2)	0·3% (0·3–0·4)	0·6% (0·5–0·7)	0·9% (0·7–1·0)	1·0% (0·9–1·2)	1·1% (1·0–1·3)
	Ovary	0·2% (0·2–0·3)	0·5% (0·4–0·6)	0·8% (0·7–0·9)	1·1% (1·0–1·3)	1·4% (1·2–1·6)	1·7% (1·5–2·0)
	Other female genital	0·1% (0·0–0·1)	0·2% (0·2–0·3)	0·4% (0·3–0·4)	0·6% (0·5–0·7)	1·0% (0·8–1·2)	1·5% (1·2–1·7)
	Colorectal	0·1% (0·1–0·2)	0·2% (0·2–0·3)	0·3% (0·3–0·4)	0·4% (0·3–0·5)	0·5% (0·4–0·6)	0·5% (0·4–0·7)
	Lung	0·1% (0·1–0·1)	0·3% (0·2–0·3)	0·7% (0·6–0·8)	1·2% (1·0–1·4)	2·2% (1·9–2·4)	2·9% (2·5–3·2)
	Melanoma	0·1% (0·1–0·1)	0·2% (0·2–0·3)	0·3% (0·2–0·4)	0·4% (0·3–0·4)	0·5% (0·4–0·6)	0·5% (0·4–0·7)
	Other	0·4% (0·3–0·4)	0·8% (0·7–0·9)	1·4% (1·2–1·5)	2·2% (2·0–2·4)	3·3% (3·0–3·6)	4·4% (4·0–4·8)
**First primary neoplasm: cervix**							
Total subsequent primary neoplasms†		1·1% (1·0–1·2)	2·7% (2·5–2·9)	4·8% (4·5–5·1)	7·8% (7·4–8·3)	11·5% (10·9–12·1)	15·8% (14·8–16·7)
	Breast	0·4% (0·3–0·5)	1·1% (1·0–1·3)	1·9% (1·7–2·1)	2·9% (2·6–3·1)	3·7% (3·3–4·0)	4·6% (4·2–5·1)
	Bladder	0·0% (0·0–0·1)	0·1% (0·1–0·2)	0·3% (0·2–0·4)	0·6% (0·4–0·7)	0·9% (0·7–1·1)	1·3% (1·0–1·6)
	Colorectal	0·1% (0·1–0·2)	0·2% (0·2–0·3)	0·5% (0·4–0·7)	1·1% (0·9–1·3)	1·8% (1·5–2·1)	2·6% (2·2–3·0)
	Lung	0·2% (0·1–0·3)	0·5% (0·4–0·6)	0·8% (0·7–1·0)	1·4% (1·2–1·6)	2·5% (2·2–2·9)	3·6% (3·2–4·2)
	Other	0·3% (0·3–0·4)	0·7% (0·6–0·9)	1·3% (1·1–1·5)	2·1% (1·8–2·3)	3·1% (2·8–3·5)	4·5% (4·0–5·1)
**First primary neoplasm: testicular**							
Total subsequent primary neoplasms‡		0·6% (0·5–0·7)	1·5% (1·3–1·6)	3·2% (2·9–3·4)	6·5% (6·0–6·9)	12·2% (11·5–13·0)	20·2% (18·9–21·5)
	Prostate	0·0% (0·0–0·0)	0·0% (0·0–0·1)	0·2% (0·1–0·3)	0·6% (0·4–0·8)	1·6% (1·3–1·9)	3·7% (3·1–4·4)
	Bladder	0·0% (0·0–0·1)	0·1% (0·1–0·2)	0·3% (0·2–0·4)	0·8% (0·6–1·0)	1·6% (1·3–1·9)	2·9% (2·4–3·6)
	Colorectal	0·1% (0·0–0·1)	0·2% (0·1–0·3)	0·4% (0·3–0·5)	0·9% (0·7–1·1)	2·0% (1·7–2·3)	3·0% (2·5–3·6)
	Lung	0·0% (0·0–0·1)	0·1% (0·1–0·2)	0·3% (0·2–0·4)	0·8% (0·7–1·0)	1·6% (1·3–2·0)	2·7% (2·2–3·2)
	Other	0·4% (0·3–0·5)	1·0% (0·9–1·2)	2·0% (1·8–2·3)	3·6% (3·2–3·9)	6·1% (5·6–6·7)	9·3% (8·4–10·2)
**First primary neoplasm: female Hodgkin lymphoma**							
Total subsequent primary neoplasms§		0·9% (0·7–1·2)	3·2% (2·8–3·7)	7·2% (6·5–8·0)	13·0% (11·9–14·0)	20·2% (18·8–21·7)	26·6% (24·7–28·6)
	Breast	0·3% (0·2–0·4)	1·3% (1·1–1·6)	3·8% (3·3–4·4)	6·7% (5·9–7·5)	10·8% (9·7–11·9)	14·4% (12·9–15·9)
	Lung	0·1% (0·0–0·2)	0·3% (0·2–0·5)	0·6% (0·4–0·9)	1·5% (1·2–2·0)	2·5% (1·9–3·1)	3·8% (3·0–4·8)
	Other	0·5% (0·4–0·7)	1·6% (1·3–1·9)	2·9% (2·5–3·4)	5·1% (4·5–5·8)	8·3% (7·3–9·3)	11·3% (10·0–12·7)
**First primary neoplasm: male Hodgkin lymphoma**							
Total subsequent primary neoplasms¶		0·6% (0·4–0·7)	1·6% (1·3–1·9)	3·4% (3·0–3·9)	6·5% (5·8–7·1)	10·7% (9·8–11·7)	16·5% (15·2–18·0)
	Lung	0·1% (0·0–0·1)	0·3% (0·2–0·5)	0·9% (0·7–1·2)	1·9% (1·5–2·3)	3·1% (2·6–3·7)	5·1% (4·3–6·0)
	Other	0·5% (0·4–0·7)	1·3% (1·0–1·5)	2·5% (2·2–2·9)	4·7% (4·2–5·3)	7·8% (7·0–8·6)	11·9% (10·7–13·2)
**First primary neoplasm: female thyroid**							
Total subsequent primary neoplasms‖		1·1% (0·8–1·4)	2·9% (2·5–3·5)	5·5% (4·7–6·2)	8·8% (7·8–9·9)	13·2% (11·8–14·8)	18·2% (16·1–20·5)
	Breast	0·5% (0·3–0·7)	1·2% (0·9–1·6)	2·4% (1·9–2·9)	4·2% (3·5–5·0)	5·9% (4·9–7·0)	8·2% (6·7–9·8)
	Other	0·6% (0·4–0·8)	1·8% (1·4–2·2)	3·1% (2·6–3·7)	4·8% (4·1–5·7)	7·6% (6·5–8·9)	10·7% (8·9–12·5)

Data are incidence (%, 95% CI). The cumulative risks for the specific subsequent primary neoplasms add up to more than the overall cumulative risk because survivors can develop more than one subsequent primary neoplasm.

* All subsequent primary neoplasms in female survivors excluding subsequent primary neoplasms of the breast.

† All subsequent primary neoplasms in female survivors excluding subsequent primary neoplasms of female genital sites.

‡ All subsequent primary neoplasms in male survivors excluding subsequent primary neoplasms of other male genital sites (prostate sites allowed).

§ All solid subsequent primary neoplasms in female survivors (excluding non-solid tumours).

¶ All solid subsequent primary neoplasms in male survivors (excluding non-solid tumours).

‖ All subsequent primary neoplasms in female survivors excluding subsequent primary neoplasms of the thyroid.

Survivors of cervical cancer had an excess risk of developing any subsequent primary neoplasm corresponding to ten excess subsequent primary neoplasms per 10 000 person-years (SIR 1·3, 95% CI 1·2–1·3; AER 10·2 per 10 000 person-years, 95% CI 8·0–12·4; table 2). SIRs for subsequent primary neoplasms of bladder, lung, and colorectal sites were statistically significantly increased. We noted a statistically significant reduction in the SIR related to breast cancer (SIR 0·8, 95% CI 0·7–0·8; table 3). The total AER of developing any subsequent primary neoplasm increased with time from cervical cancer diagnosis to an AER of 32·3 per 10 000 person-years (95% CI 15·4–49·1) subsequent to 30 years from diagnosis (p<0·0001), as did the AER for developing lung, colorectal, and bladder cancer (each p<0·0001; table 4). In patients who had survived at least 30 years, the AERs for lung cancer (17·2 per 10 000 person-years, 95% CI 8·8–25·6), colorectal cancer (10·7, 3·5–17·9), and bladder cancer (10·1, 4·5–15·7) accounted for 37%, 23%, and 22% of the total number of excess neoplasms, respectively (total AER 46·5 per 10 000 person-years when negative AERs are excluded). The cumulative incidences of subsequent lung, colorectal, and bladder neoplasms at 35 years from diagnosis were 3·6% (95% CI 3·2–4·2), 2·6% (2·2–3·0), and 1·3% (1·0–1·6), whereas incidences of 1·6%, 1·4%, and 0·4% were expected, respectively (figure, table 5).

Survivors of testicular cancer had an excess risk of developing any subsequent primary neoplasm corresponding to 19 excess subsequent primary neoplasms per 10 000 person-years (SIR 1·8, 95% CI 1·7–1·9; AER 18·9 per 10 000 person-years, 95% CI 16·6–21·1; table 2). SIRs for subsequent primary neoplasms of bladder, colorectal, lung, and prostate sites were statistically significantly increased (table 3). The total AER of developing any subsequent primary neoplasm increased with time from testicular cancer diagnosis to an AER of 127·0 per 10 000 person-years (95% CI 100·0–154·0) subsequent to 30 years from diagnosis (p<0·0001), as did the AER for developing bladder, colorectal, lung, and prostate cancer (each p<0·0001; table 4). In patients who had survived at least 30 years, the AERs for prostate cancer (25·3 per 10 000 person-years, 95% CI 12·1–38·6), bladder cancer (22·8, 13·0–32·7), colorectal cancer (19·6, 9·2–29·9), and lung cancer (9·5, 0·1–18·8) accounted for 20%, 18%, 15%, and 8% of the total number of excess neoplasms, respectively. The cumulative incidences of subsequent primary neoplasms at 35 years from diagnosis were 3·7% (95% CI 3·1–4·4) for prostate, 2·9% (95% CI 2·4–3·6) for bladder, 3·0% (2·5–3·6) for colorectal, and 2·7% (2·2–3·2) for lung, whereas incidences of 2·9%, 1·1%, 1·8%, and 2·0% were expected, respectively (figure, table 5).

Female survivors of Hodgkin lymphoma had an excess risk of developing any subsequent primary neoplasm corresponding to 56 excess subsequent primary neoplasms per 10 000 person-years (SIR 3·1, 95% CI 2·9–3·3; AER 55·7 per 10 000 person-years, 95% CI 50·4–61·1; table 2). SIRs for subsequent primary cancers of breast and lung sites were statistically significantly increased (table 3). The total AER of developing any subsequent primary neoplasm increased with time from Hodgkin lymphoma diagnosis to an AER of 168·6 per 10 000 person-years (95% CI 129·5–207·8) subsequent to 30 years from diagnosis (p<0·0001), as did the AER for developing breast and lung cancer (each p<0·0001; table 4). In patients who had survived at least 30 years, the AERs for breast cancer (71·8 per 10 000 person-years, 95% CI 46·4–97·2) and lung cancer (26·0, 11·2–40·8) accounted for 43% and 15% of the total number of excess neoplasms, respectively. The cumulative incidences of breast and lung neoplasms at 35 years from diagnosis were 14·4% (95% CI 12·9–15·9) and 3·8% (3·0–4·8), whereas incidences of 4·9% and 0·9% were expected, respectively (figure, table 5).

Male survivors of Hodgkin lymphoma had an excess risk of developing any subsequent primary neoplasm corresponding to 30 excess subsequent primary neoplasms per 10 000 person-years (SIR 2·6, 95% CI 2·4–2·8; AER 29·9 per 10 000 person-years, 95% CI 26·3–33·6; table 2). The SIR for a subsequent primary lung cancer was statistically significantly increased (table 3). The total AER of developing any subsequent primary neoplasm increased with time from Hodgkin lymphoma diagnosis to an AER of 121·9 per 10 000 person-years (95% CI 91·3–152·4) subsequent to 30 years from diagnosis (p<0·0001), as did the AER for developing lung cancer (p<0·0001; table 4). In patients who had survived at least 30 years, the AER for lung cancer (50·2 per 10 000 person-years, 95% CI 33·0–67·3) accounted for 41% of the total number of excess neoplasms. The cumulative incidence of lung neoplasms in male survivors of Hodgkin lymphoma was 5·1% (95% CI 4·3–6·0) at 35 years from diagnosis, whereas an incidence of 1·4% was expected (figure, table 5).

Female survivors of thyroid cancer had an excess risk of developing any subsequent primary neoplasm corresponding to 13 excess subsequent primary neoplasms per 10 000 person-years (SIR 1·4, 95% CI 1·2–1·5; AER 13·1 per 10 000 person-years, 95% CI 8·4–17·8; table 2). The SIR for a subsequent primary breast cancer was statistically significantly increased (table 3). The total AER of developing any subsequent primary neoplasm increased with time from thyroid cancer diagnosis to an AER of 21·9 per 10 000 person-years (95% CI −10·9 to 54·7) subsequent to 30 years from diagnosis, (p=0·03; table 4). The cumulative incidence of all subsequent primary neoplasms in female survivors of thyroid cancer was 18·2% (95% CI 16·1–20·5) at 35 years from diagnosis (table 5), whereas an incidence of 13·6% was expected. The cumulative incidence of breast cancer was 8·2% (6·7–9·8) at 35 years from diagnosis, whereas an incidence of 6·0% was expected (figure; table 5).

Survivors of ovarian cancer had an excess risk of developing any subsequent primary neoplasm corresponding to 12 excess subsequent primary neoplasms per 10 000 person-years (SIR 1·4, 95% CI 1·2–1·5; AER 12·3 per 10 000 person-years, 95% CI 7·5–17·1; table 2). A statistically significant reduction was found in the SIR for the development of a subsequent primary breast cancer (0·8, 95% CI 0·6–0·9; table 3). The cumulative incidence of all subsequent primary neoplasms was 13·9% (95% CI 12·2–15·7) at 35 years from diagnosis (table 2), whereas an incidence of 12·3% was expected.

Both male and female survivors of CNS tumours had an excess risk of developing any subsequent primary neoplasm corresponding to 15 and 11 excess neoplasms, respectively (male survivors: SIR 1·7 [95% CI 1·5–1·9]; AER 14·8 per 10 000 person-years [95% CI 10·7–19·0]; female survivors: SIR 1·3 [1·2–1·5]; AER 11·1 [7·0–15·2]). A statistically significant reduction in the SIR for the development of a subsequent primary breast cancer was found (SIR 0·7, 95% CI 0·6–0·8; table 3). The cumulative incidence of all subsequent primary neoplasms was 11·9% (95% CI 10·6–13·3) in female survivors of CNS tumours and 10·0% (8·7–11·4) in male survivors of CNS tumours at 35 years from diagnosis (table 2), whereas an incidence of 9·4% was expected.

Sensitivity analyses showed that inclusion of leukaemia after AYA Hodgkin lymphoma, non-Hodgkin lymphoma, and leukaemia had little effect on the SIRs and AERs (appendix p 10). Inclusion of sarcomas after AYA soft-tissue sarcoma and bone tumour increased the AERs, but there was substantial overlap in confidence intervals (appendix p 10).

## Discussion

We show that the excess number of subsequent primary neoplasms observed increases with increased period of follow-up from diagnosis after each AYA cancer investigated. In patients who had survived at least 30 years from diagnosis of cervical cancer, testicular cancer, Hodgkin lymphoma in women, breast cancer, and Hodgkin lymphoma in men, we identified just a small number of specific subsequent primary neoplasms that account for 82%, 61%, 58%, 45%, and 41% of the total excess number of neoplasms, respectively. To our knowledge, our study is the first to report excess risks of specific types of subsequent primary neoplasms after each of 16 types of AYA cancer. One study has previously addressed the risk of all subsequent primary neoplasms combined after each AYA cancer,^3^ but no study has previously considered specific subsequent primary neoplasms.

In our study, the burden of the excess number of neoplasms beyond 30 years from diagnosis accounted for by lung cancer was substantial and apparent across all AYA cancers investigated (breast, cervical, testicular, and Hodgkin lymphoma in males and females). It is well known that smoking is carcinogenic and the main risk factor for developing lung cancer. Additionally, smoking increases the risk of developing subsequent primary neoplasms, particularly oral or pharyngeal, oesophageal, stomach, lung, and haematological malignancies.^21^ Kaul and colleagues^22^ reported that 33% of survivors of AYA cancer were current smokers compared with 22% in a non-cancer comparison group matched on age, sex, and other factors. This finding confirmed earlier work that AYA cancer survivors smoke in excess.^23^ Young female survivors have a higher risk of being a current smoker compared with young male survivors, and this difference is largely accounted for by cervical cancer.^24^ Consistent with our findings, Underwood and colleagues^25^ reported that survivors of cervical cancer have a two-fold increased risk of developing smoking-related cancers compared with the general population. Studies have shown that smoking is a risk factor for developing lung cancer in survivors of breast cancer^26^ and Hodgkin lymphoma,^27^ and that this risk is further enhanced in patients who have been treated with radiotherapy (for breast cancer and Hodgkin lymphoma) and chemotherapy (for Hodgkin lymphoma). We observed a decrease in the number of excess lung cancers in male survivors of Hodgkin lymphoma with more recent diagnosis. Although this decrease could be caused by a number of factors, it might be related to a change in smoking habits in more recent decades (ie, a reduction in male smokers). The evidence presented in our study, along with previous literature on smoking in cancer survivors, clearly suggests that clinical follow-up of survivors of AYA cancer, particularly survivors of breast cancer, cervical cancer, and Hodgkin lymphoma, should focus on subsequent lung cancer and provision of smoking cessation advice.

Generally, it is difficult to compare risks of subsequent primary neoplasms between survivors of AYA and childhood cancer because there are so many important confounding influences. However, lung cancer as a subsequent primary neoplasm is an exception to this general rule in that from our population-based national cohort of survivors of childhood cancer in Britain, we reported that in survivors aged 40 years and older, lung cancer was associated with an AER of only 2·9 per 10 000 person-years (95% CI 0·4–5·5), which accounted for just 9% of the total AER.^28^ By contrast, in the present analysis, the AER for lung cancer after at least 30 years from diagnosis of AYA cancer was substantially higher and accounted for a much greater proportion of the total AER. Notably, by contrast with survivors of AYA cancer, the odds ratio for being a current regular smoker among survivors of childhood cancer in Britain was 0·51 compared with the general population of Britain.^29^

In female survivors of breast cancer, the SIRs reported in our study were broadly consistent with those reported in previous literature.^5^, ^6^, ^7^ The increased risk of ovarian cancer could relate to shared hormonal and genetic (eg, *BRCA1* and *BRCA2* mutations) risk factors.^30^ The increased risk of uterine cancers might relate to partial oestrogen agonists used to treat the breast cancer—a previous large case-control study found that risk of uterine cancer increases with duration of tamoxifen treatment.^31^ Of the six anatomical sites at which an excess of subsequent primary neoplasms was observed, only the lungs would be directly exposed if external-beam radiotherapy was used to treat the breast cancer. A previous large case-control study showed a dose-response relation between radiotherapy and risk of lung cancer in breast cancer survivors diagnosed at any age (not AYA-specific).^26^ Existing literature suggests that chest radiotherapy and smoking are both likely contributors to the substantial number of excess neoplasms accounted for by lung cancer.

The bladder and bowel would be directly exposed if external-beam radiotherapy was used to treat cervical cancer. A large case-control study showed a dose-response relation between radiotherapy and the risk of both bladder and rectal cancers in cervical cancer survivors.^32^ Existing literature suggests that pelvic irradiation and smoking are likely contributors to the number of excess neoplasms accounted for by lung, colorectal, and bladder cancer. Clinical follow-up of survivors of AYA cervical cancer, particularly where pelvic irradiation is used, should focus on lung, bowel, and bladder cancers.

Treatment for testicular cancer can involve irradiating the para-aortic lymph nodes, which might explain the excess of subsequent primary neoplasms seen in abdominal sites (prostate, bladder, and colorectal). The excess of subsequent primary neoplasms observed in the abdomen is consistent with international studies of testicular cancer survivors.^8^ The excess of lung subsequent primary neoplasms might be caused by radiotherapy to the lungs, since previous studies have reported an increased risk of lung cancer in survivors of testicular cancer who were given chest radiotherapy.^8^ Clinical follow-up of survivors of AYA testicular cancer should focus on prostate, bladder, colorectal, and lung cancers.

The lungs would be directly exposed if external-beam radiotherapy was used to treat Hodgkin lymphoma; previous studies of Hodgkin lymphoma survivors have provided evidence of a dose-dependent increase in lung cancer risk with radiotherapy with or without chemotherapy.^27^ Our findings are consistent with previous large-scale studies of female survivors of Hodgkin lymphoma, for which a substantial amount of literature already exists, and by contrast with other AYA cancers considered here, we have little to add.^9^, ^10^, ^11^ Our findings support the decrease in lung cancer risk with more recent calendar period of diagnosis that was reported in a Dutch study of Hodgkin lymphoma survivors.^9^ This decrease might be due to a latency effect, where more recently diagnosed survivors simply have not had enough time to develop a lung subsequent primary neoplasm, or it might be caused by changes in treatment for Hodgkin lymphoma during recent decades, including withholding radiotherapy or improvements in the delivery of radiotherapy resulting in less damage to healthy tissue than in previous decades.^33^ However, because a decrease in lung cancer risk was not seen after Hodgkin lymphoma in women, it is possible that the decrease in lung cancer in men is caused by other environmental influences, such as changes in smoking habits. Previous studies have reported an increase in the number of excess lung cancers with increasing years from diagnosis;^9^, ^10^ however, to our knowledge, our study is the first to report the number of excess lung cancers in male and female survivors separately. Existing literature suggests that treatment (radiotherapy and chemotherapy), in addition to smoking, could contribute to the number of excess neoplasms accounted for by lung cancer.^9^ Clinical follow-up of male survivors of AYA Hodgkin lymphoma should focus on lung cancer and provision of smoking cessation advice.

Younger age at radiation exposure is a risk factor for the development of breast cancer in many populations exposed to radiation, including atomic bomb survivors, patients with tuberculosis monitored with x-rays, and children with benign disorders treated with radiotherapy.^34^ Thus, the effect of age at diagnosis (closely related to age at radiotherapy) of Hodgkin lymphoma on the risk of breast cancer in our cohort is not surprising.

Knowledge of late effects of cancer treatment has resulted in lower radiation exposures for treatments of good prognosis cancers in recent years;^35^ however, the multivariable regression showed that the risk of developing a subsequent primary neoplasm did not vary with decade of diagnosis of AYA cancer apart from lung cancer after Hodgkin lymphoma in males. Therefore, currently there is little evidence of a detectable impact.

Until recently, no internationally agreed clinical guidelines existed regarding surveillance for specific types of neoplasm after AYA cancer, but this is now being addressed by the International Late Effects of Childhood Cancer Guideline Harmonization Group.^36^ So far, two such guidelines have been published.^37^, ^38^ There are also guidelines in development relating to second primary CNS tumours and second primary bowel cancers.

Strengths of our cohort study relate to its large scale and population-based design, with the inclusion of all 5-year survivors of AYA cancer in England and Wales. The study design minimises selection bias and the results are generalisable to the English and Welsh population. Our study had much greater statistical power than the only comparable previous study by Lee and colleagues,^3^ because we report almost double the number of subsequent primary neoplasms and an additional million person-years of follow-up. Most previous studies investigating the risk of subsequent primary neoplasms with years from diagnosis have mainly focused on the SIR, a measure of multiplicative risk that relates to an unspecified baseline risk and is therefore difficult to interpret. We concentrated on the AER, which is the excess number of subsequent primary neoplasms beyond those expected from the general population, and so is directly interpretable in terms of adverse health impact on survivors. To our knowledge, our study is the first to report AERs by years from diagnosis for each specific AYA cancer.

A limitation of using cancer registration data is the absence of detailed treatment information. Treatment for AYA cancer varies greatly by cancer type; therefore, in the absence of treatment data, we chose to determine risks in relation to specific cancer types. However, inevitably there is variation in the intensity of treatment given for a specific type of cancer, depending on the stage at diagnosis and whether the disease recurs or relapses after initial treatment. Crude treatment information is inherent in cohort studies based on cancer registry data; however, we are planning to conduct case-control studies with detailed treatment dosimetry, questionnaires for lifestyle and other relevant factors, and saliva collection for genotypic factors. A potential limitation of our study is that our results might not be generalisable to populations outside of England and Wales.

Another limitation is the possibility that recurrence or metastases of the AYA cancer could have been mistaken for a subsequent primary neoplasm. However, we used the IACR/IARC rules to define multiple primary cancers and further excluded any additional neoplasms close to the AYA cancer site. By using these criteria, we excluded any contralateral subsequent primary neoplasms such as breast, kidney, ovarian, lung, or testicular subsequent primary neoplasms. Thus, our estimate of the risk of specific subsequent primary neoplasms after specific AYA cancers is probably conservative, and although many of the estimates we report are substantial, they are likely to be an underestimate of the true risk.

In conclusion, our data show that the excess number of subsequent primary neoplasms observed increases with increased period of follow-up from diagnosis after each AYA cancer investigated. In patients who had survived at least 30 years from diagnosis of cervical cancer, testicular cancer, Hodgkin lymphoma, and breast cancer, we identified just a small number of specific subsequent primary neoplasms that account for high proportions of the total excess number of neoplasms. A notable finding was the excess number of neoplasms accounted for by lung cancer across all AYA groups investigated in detail, in addition to subsequent primary neoplasms occurring in potentially irradiated sites. Our findings provide an evidence base for clinical follow-up relating specifically to the AYA population.
