# Supplementary material for: Risk of subsequent primary neoplasms in survivors of adolescent and young adult cancer (Teenage and Young Adult Cancer Survivor Study): a population-based, cohort study
Source: Lancet Oncol. 2019 Apr;20(4):531–45. doi: 10.1016/S1470-2045(18)30903-3 (PMC6494975; doi:10.1016/S1470-2045(18)30903-3)
Supplement: Supplementary appendix [file mmc1.pdf]

THE LANCET
Oncology

Supplementary appendix

This appendix formed part of the original submission and has been peer reviewed. We post it as supplied by the authors.

Supplement to: Bright C J, Reulen R C, Winter D L, et al. Risk of subsequent primary neoplasms in survivors of adolescent and young adult cancer (Teenage and Young Adult Cancer Survivor Study): a population-based, cohort study. *Lancet Oncol* 2019; published online Feb 20. http://dx.doi.org/10.1016/S1470-2045(18)30903-3.

Risk of subsequent primary neoplasms in survivors of adolescent and young adult cancer: The Teenage and Young Adult Cancer Survivor Study – a population-based cohort study

Corresponding Author: Michael M Hawkins. Email: m.m.hawkins@bham.ac.uk

Web appendix

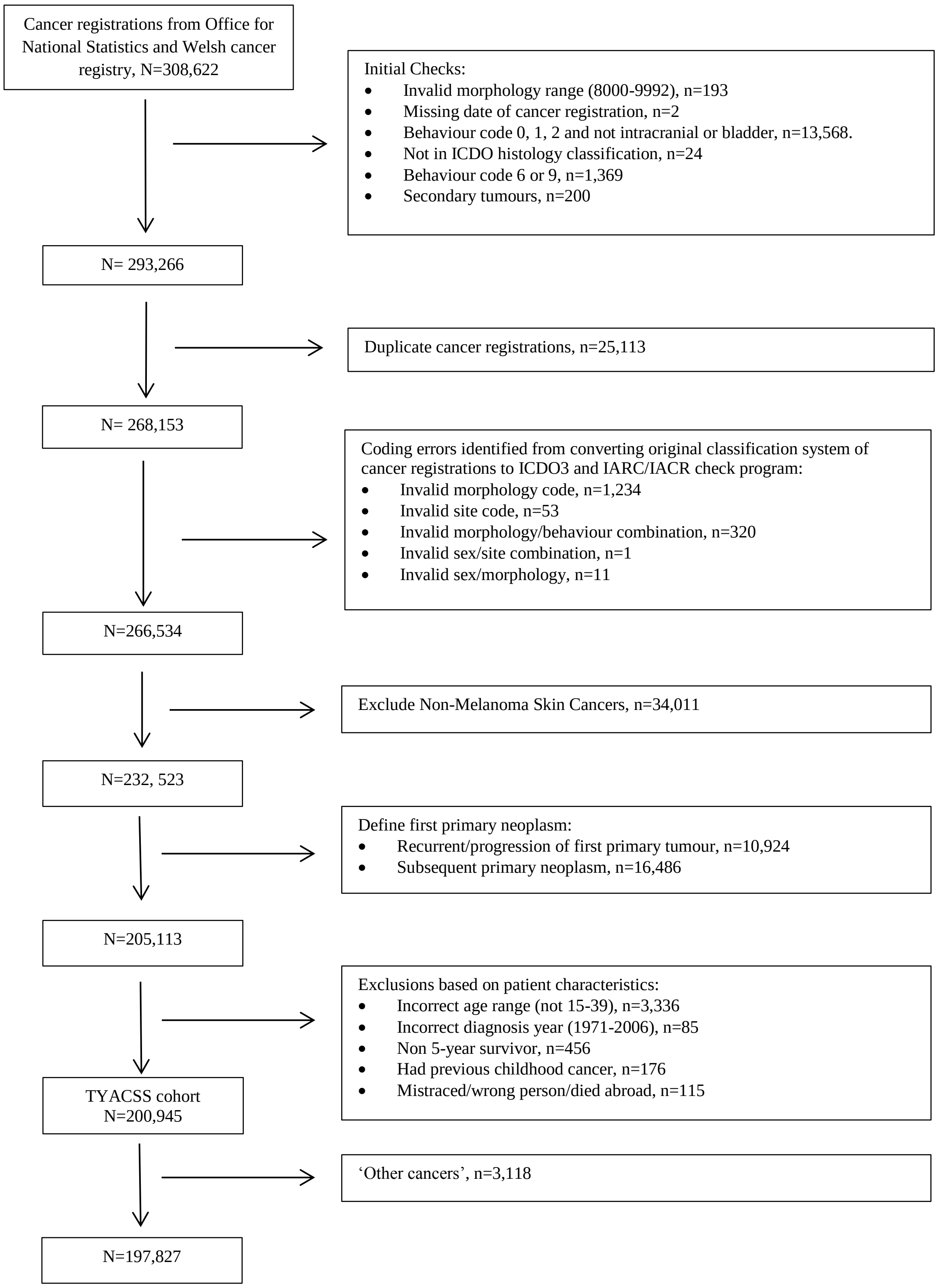

Appendix Figure 1: Process to create the Teenage and Young Adult Cancer Survivor Study (TYACSS) cohort from cancer registration data.

Appendix Table 1: Groupings of AYA cancer based on the adolescent and young adult cancer classification scheme. (Refinements are highlighted in red)

Broader Groupings used in current study	AYA classification	AYA code
**Leukaemia**	Acute Lymphoid Leukaemia	1.1
	Acute Myeloid Leukaemia	1.2
	Chronic Myeloid Leukaemia	1.3
	Other and Unspecified Leukaemias	1.4
**Non-Hodgkin Lymphoma**	Non- Hodgkin Lymphoma, specified subtype	2.1.1
	Unspecified Non-Hodgkin Lymphoma	2.1.2
	Myeloma, mast cell tumours and miscellaneous lymphoreticular neoplasms NEC	9.2.3
**Hodgkin Lymphoma**	Hodgkin Lymphoma, specified subtype	2.2.1
	Hodgkin lymphoma NOS	2.2.2
**CNS Intracranial (Including Brain, Meninges and Pituitary Gland)**	Pilocytic Astrocytoma	3.1.1
	Other specified low grade astrocytic tumours	3.1.2
	Glioblastoma and anaplastic astrocytoma	3.1.3
**Spinal Cord & other CNS**	Astrocytoma, NOS	3.1.4
	Oligodendroglioma	3.2.1
	Other specified glioma	3.2.2
	Glioma, NOS	3.2.3
	Ependymoma	3.3
	Medulloblastoma	3.4.1
	Supratentorial PNET	3.4.2
	Craniopharyngioma	3.5.1
	Other pituitary tumours	3.5.2
	Pineal tumours	3.5.3
	Choroid plexus tumours	3.5.4
	Meningioma	3.5.5
	Nerve sheath tumours of CNS	3.5.6
	Other specified intracranial and intraspinal neoplasms	3.5.7
	Unspecified malignant intracranial and intraspinal neoplasms	3.6.1
	Unspecified benign and borderline intracranial and intraspinal neoplasms	3.6.2
	Germ cell intracranial	6.2.1
**Bone Neoplasms**	Osteosarcoma	4.1
	Chondrosarcoma	4.2
	Ewing sarcoma of bone	4.3.1
	Ewing sarcoma of specified site other than bone	4.3.2
	Ewing sarcoma of unspecified site	4.3.3
	Other specified bone tumours	4.4.1
	Unspecified bone tumours	4.4.2

**Soft Tissue Sarcoma**	Fibrosarcoma	5.1.1
	Malignant Fibrous Histiocytoma	5.1.2
	Dermatofibrosarcoma	5.1.3
	Rhabdomyosarcoma	5.2
	Liposarcoma	5.3.1
	Leiomyosarcoma	5.3.2
	Synovial sarcoma	5.3.3
	Clear cell sarcoma	5.3.4
	Blood vessel tumours	5.3.5
	Nerve sheath tumours	5.3.6
	Alveolar soft part sarcoma	5.3.7
	Other specified	5.3.8
	Unspecified soft tissue sarcoma	5.4
**Melanoma**	Melanoma	7.1
**Carcinomas – by site**		8
**Thyroid**	Thyroid Carcinoma	8.1
**Head and Neck**	Nasopharyngeal carcinoma	8.2.1
	Other sites in lip, oral cavity and pharynx	8.2.2
	Nasal cavity, middle ear, sinuses, larynx and other and ill-defined head and neck	8.2.3
**Lung**	Trachea, bronchus and lung	8.3
**Breast**	Carcinoma of breast (women only)	8.4
**Bladder**	Carcinoma of bladder	8.5.2
	Unspecified malignant neoplasms, NEC (in bladder site)	10
	Unspecified benign and borderline neoplasms, NEC (in bladder site)	Not in AYA
	Benign and borderline neoplasms of bladder (ICDO3 code 8010-8589, 8982)	Not in AYA
**GU Tract (other)**	Carcinoma of kidney	8.5.1
	Carcinoma of other and ill-defined sites in GU tract	8.5.5
	Wilms tumour	9.1.1
**Ovary**	Carcinoma of ovary	8.5.3
	Germ cell and trophoblastic neoplasms of gonads (women only)	6.1
	Other specified gonadal tumours (women only)	9.2.2
**Cervix**	Carcinoma of cervix	8.5.4
**Gastrointestinal Tract**	Carcinoma of colon and rectum	8.6.1
	Carcinoma of stomach	8.6.2

	Carcinoma of liver and intrahepatic bile ducts	8.6.3
	Carcinoma of pancreas	8.6.4
	Carcinoma of other and ill-defined sites in GI tract	8.6.5
**Testis**	Germ cell gonadal (men only)	6.1
	Other specified gonadal tumours (men only)	9.2.2
**Other**	Other non-gonadal sites	6.2.2
	Non melanoma skin cancer (non-epithelial tumours only)	7.2
	Carcinoma of breast (men only)	8.4
	Adrenocortical carcinoma	8.7.1
	Carcinoma of other and ill-defined sites, NEC	8.7.2
	Neuroblastoma	9.1.2
	Other paediatric and embryonal, NEC	9.1.3
	Paraganglioma and glomus	9.2.1
	Other specified neoplasms, NEC	9.2.4
	Unspecified malignant neoplasms, NEC	10

Abbreviations: AYA= adolescents and young adults; NEC=not elsewhere classified; NOS= not otherwise specified; GI= gastrointestinal; GU=genitourinary; CNS= central nervous system; PNET= primitive neuroectodermal tumour.

Appendix Table 2: Description of 'Other' AYA cancers excluded from analysis.

Description	Number of Subsequent Primary Neoplasms
CNS tumour (located outside of the CNS)	62
Germ cell neoplasm (non-gonadal, extracranial)	607
Non-epithelial Skin Cancer	192
Breast neoplasm in men	103
Adrenocortical carcinoma	82
Carcinoma of other and ill-defined sites, NEC	528
Neuroblastoma	59
Other paediatric and embryonal neoplasms, NEC	63
Paraganglioma and glomus neoplasms	143
Other specified neoplasms, NEC	664
Unspecified malignant neoplasm, NEC	615
Total	3,118

Abbreviations:, CNS – central nervous system, NEC – not elsewhere classified

Appendix Table 3: Exclusions of AYA cancer and subsequent primary neoplasms combinations

**Subsequent Primary Neoplasms**	**First Primary Neoplasm**											
	**Breast (women)**	**Testis**	**Cervix**	**Melanoma**	**Hodgkin lymphoma**	**NHL**	**Thyroid**	**Brain**	**Colorectal**	**STS**	**Ovary**	**Leukaemia**
Breast (women)	Excluded	N/A										
Corpus Uteri			Excluded								Excluded	
Ovary		N/A	Excluded								Excluded	
Other Genital (women)		N/A	Excluded								Excluded	
Kidney												
Bladder												
Other Urinary												
Prostate	N/A		N/A								N/A	
Other Genital (men)	N/A	Excluded	N/A								N/A	
Colorectal									Excluded			
Oesophagus												
Stomach												
Pancreas												
Other Digestive[1]												
Lung & Bronchus												
Other Respiratory												
Melanoma				Excluded								
Brain								Excluded				
Meninges												
Spinal Cord & other CNS												
Pituitary Gland												
Thyroid							Excluded					
STS										Excluded		
Bone Sarcoma										Excluded		
NHL					Excluded	Excluded						Excluded
Leukaemia					Excluded	Excluded						Excluded
Oral												
Other												
--Hodgkin lymphoma					Excluded	Excluded						Excluded
--Other lymphoid					Excluded	Excluded						Excluded
--Eye				Excluded								
--Breast (Men only)	N/A											
--Other Endocrine												

Appendix Table 3 continued

Subsequent Primary Neoplasms	First Primary Neoplasms										
	**Bladder**	**Pituitary gland**	**Head & Neck**	**Lung**	**Other Genital (women)**	**Spinal Cord and Other CNS**	**Other Digestive**	**Meninges**	**Urinary (other)**	**Bone Tumour**	**Other Genital (men)**
Breast (women)											N/A
Corpus Uteri					Excluded						N/A
Ovary					Excluded						N/A
Other Genital (women)					Excluded						N/A
Kidney	Excluded								Excluded		
Bladder	Excluded								Excluded		
Other Urinary	Excluded								Excluded		
Prostate					N/A						Excluded
Other Genital (men)					N/A						Excluded
Colorectal											
Oesophagus							Excluded				
Stomach							Excluded				
Pancreas							Excluded				
Other Digestive							Excluded				
Lung & Bronchus				Excluded							
Other Respiratory			Excluded[2]	Excluded							
Melanoma											
Brain											
Meninges								Excluded			
Spinal Cord & other CNS						Excluded					
Pituitary Gland		Excluded									
Thyroid											
STS										Excluded	
Bone Sarcoma										Excluded	
NHL											
Leukaemia											
Oral			Excluded								
Other											
--Hodgkin lymphoma											
--Other lymphoid											
--Eye											
--Breast (Men only)					N/A						
--Other Endocrine											

Abbreviations: NHL – non Hodgkin lymphoma, STS – soft tissue sarcoma, CNS – central nervous system.

[1] All liver subsequent primary neoplasms were excluded because of substantial likelihood of metastatic spread.

[2] Nasal cavity, middle ear, accessory sinuses and larynx excluded only. Trachea, lung and bronchus not excluded

Appendix Table 4: Absolute Excess Risks (AERs) and Relative Excess Risks (RERs) of subsequent primary breast neoplasms among female survivors of Hodgkin lymphoma by age at diagnosis of Hodgkin lymphoma

	Obs/Ex	AER (95% CI)	RER (95% CI)[1]
**Overall**	431/136·3	26·7 (23·0,30·4)	
**Age at Diagnosis**			
15-19	132/12·2	54·4 (44·2,64·7)	1·0 (1·0,1·0)
20-24	115/26·2	29·4 (22·4,36·3)	0·5 (0·4,0·7)
25-29	76/31·2	18·1 (11·2,25·0)	0·4 (0·2,0·6)
30-34	63/34·0	15·2 (7·1,23·4)	0·3 (0·2,0·6)
35-39	45/32·6	8·7 (-0·5,17·9)	0·2 (0·1,0·5)
p-trend		*<0·0001*	*<0·0001*

Abbreviations: Obs- observed number of subsequent primary neoplasms, Ex- expected number of subsequent primary neoplasms, AER-absolute excess risk, RER – relative excess risk, CI- confidence interval
[1]From an externally controlled Poisson regression model which contained the following factors: attained age, decade of diagnosis and age at diagnosis.

Appendix Table 5: Absolute Excess Risks (AERs) and Relative Excess Risks (RERs) of subsequent primary lung neoplasms among male survivors of Hodgkin lymphoma by decade of diagnosis of Hodgkin lymphoma

	Obs/Ex	AER (95% CI)	RER (95% CI)[1]
**Overall**	198/41·3	10·9 (9·0,12·8)	
**Decade of Diagnosis**			
1971-1979	110/23·4	18·9 (14·4,23·3)	1·0 (1·0,1·0)
1980-1989	78/14·1	11·9 (8·7,15·1)	0·9 (0·6,1·3)
1990-2006	10/3·9	1·4 (-0·0,2·8)	0·2 (0·1,0·5)
p-trend		*<0·0001*	*0·0001*

Abbreviations: Obs- observed number of subsequent primary neoplasms, Ex- expected number of subsequent primary neoplasms, AER-absolute excess risk, RER – relative excess risk, CI- confidence interval
[1]From an externally controlled Poisson regression model which contained the following factors: attained age, decade of diagnosis and age at diagnosis.

Appendix Table 6: Comparison of SIRs and AERs in Table 1 with SIRs and AERs calculated allowing leukaemia as an subsequent primary neoplasm after AYA Hodgkin lymphoma, non-Hodgkin lymphoma and leukaemia; and allowing any sarcoma as a subsequent primary neoplasm after AYA soft tissue sarcoma and bone tumours

First Primary Neoplasm	Results from Table 1 or sensitivity analysis	Number of SPN	Females			Males		
			Obs/Ex	SIR (95% CI)	AER (95% CI)	Obs/Ex	SIR (95% CI)	AER (95% CI)
Hodgkin Lymphoma	Table 1	1606	903/288·2	3·1 (2·9,3·3)	55·7 (50·4,61·1)	703/271·9	2·6 (2·4,2·8)	29·9 (26·3,33·6)
	Sensitivity	1665	928/293.0	3.2 (3.0,3.4)	57.6 (52.1,63.0)	737/281.7	2.6 (2.4,2.8)	31.6 (27.9,35.3)
Non-Hodgkin Lymphoma	Table 1	511	216/149·5	1·4 (1·3,1·7)	14·8 (8·4,21·2)	295/163·4	1·8 (1·6,2·0)	18·6 (13·8,23·4)
	Sensitivity	529	221/151.9	1.5 (1.3,1.7)	15.3 (8.9,21.8)	308/168.9	1.8 (1.6,2.0)	19.7 (14.8,24.5)
Leukaemia	Table 1	234	120/63·3	1·9 (1·6,2·3)	22·9 (14·2,31·5)	114/44·4	2·6 (2·1,3·1)	22·7 (15·9,29·5)
	Sensitivity	237	121/64.3	1.9 (1.6,2.2)	22.9 (14.2,31.6)	116/46.1	2.5 (2.1,3.0)	22.8 (15.9,29.7)
Soft Tissue Sarcoma	Table 1	400	255/165·7	1·5 (1·4,1·7)	19·8 (12·9,26·8)	145/106·5	1·4 (1·1,1·6)	9·3 (3·6,15·0)
	Sensitivity	428	269/166.8	1.6 (1.4,1.8)	22.7 (15.6,29.9)	159/107.8	1.5 (1.3,1.7)	12.4 (6.4,18.4)
Bone Tumour	Table 1	100	52/32·7	1·6 (1·2,2·1)	14·7 (3·9,25·5)	48/33·1	1·4 (1·1,1·9)	8·1 (0·7,15·6)
	Sensitivity	113	55/33.0	1.7 (1.3,2.2)	16.8 (5.7,27.9)	58/33.6	1.7 (1.3,2.2)	13.4 (5.2,21.5)

Abbreviations: Obs- observed number of subsequent primary neoplasms, Ex- expected number of subsequent primary neoplasms, SIR- standardised incidence ratio, AER- absolute excess risk, CI- confidence interval
